# Investigating the Effect of Lapatinib on the Performance of Sonodynamic Therapy Against Prostate Cancer via Inhibition of the ABCG2 Efflux System

**DOI:** 10.1002/prp2.70340

**Published:** 2026-10-22

**Authors:** Yidong Wang, Hamzah Masood, Nikolitsa Nomikou

**Affiliations:** ^1^ Division of Surgery and Interventional Science Faculty of Medical Sciences University College London London UK

**Keywords:** ABCG2 transporter, drug efflux, lapatinib, prostate cancer, sensitizer, sonodynamic therapy

## Abstract

Sonodynamic therapy (SDT) is an emerging site specific and minimally‐invasive modality for cancer treatment. Its efficacy heavily relies on the accumulation and retention of systemically‐administered sensitizing agents within tumor cells. Active molecular efflux via membrane transporters, such as the human ATP‐binding cassette (ABC) superfamily G member 2 (ABCG2) transporter, can pose a major challenge by reducing intracellular levels of sensitizers and hinder sonodynamic activity. In this study, the role of ABCG2 transporter in the retention of nanoparticulate hematoporphyrin was investigated using prostate cancer cells. The potential of lapatinib to improve cellular retention of the sensitizer via inhibition of ABCG2 and improve the efficacy of SDT was evaluated. The effect of hypoxia and neuroendocrine differentiation on ABCG2 activity and the role of lapatinib were examined. Results showed that hypoxia intensifies ABCG2 localization in the cytoplasmic membrane. There was no effect of lapatinib on the cellular accumulation of nanoparticulate hematoporphyrin during exposure of the cell systems to the sensitizer. Following sensitizer withdrawal, a sharp intracellular hematoporphyrin level decrease was observed within the first 2 h. Lapatinib significantly attenuated the reduction of intracellular sensitizer in hypoxia, during the first 8 h after withdrawal of the sensitizer. Lapatinib can extend the intracellular presence of the sonosensitizer, prolonging the therapeutic window for performing SDT. This outcome underlined the value of lapatinib for improving the sensitizer intracellular levels in hypoxic areas of the tumor mass, where sensitizer supply is expected to be low, due to the low vascularization levels.

**Abbreviations**
ABCG2: ATP‐binding cassette superfamily G member 2
ADT: androgen deprivation therapy
ALA: 5‐aminolevulinic acid
EGFR: epidermal growth factor receptor
HER2: human epidermal growth factor receptor 2
HP: hematoporphyrin
HPNP(s): hematoporphyrin‐containing polymer‐based nanoparticle(s)
NEPC: neuroendocrine differentiated PC3
PDT: photodynamic therapy
ROS: reactive oxygen species
SDT: sonodynamic therapy
US: ultrasound

## 1 Introduction

Sonodynamic therapy (SDT) is emerging as an innovative treatment approach that shows significant potential in eliminating solid tumors [1, 2]. SDT employs sensitizers (sonosensitizers), like porphyrins, in combination with low‐intensity ultrasound for the generation of cytotoxic reactive oxygen species (ROS) and the subsequent site‐specific ablation of solid tumors. The sonosensitizer and ultrasound (US) exposure are both non‐toxic and only induce therapeutic effects when combined. Compared to similar alternative approaches, such as photodynamic therapy (PDT), where light is applied to activate the production of ROS in the presence of a sensitizer, ultrasound has the attribute of deep tissue penetration, it can be applied in a non‐invasive or minimally‐invasive manner, and thus offers better therapeutic performance for non‐superficial lesions [3, 4]. Due to its features, SDT has been suggested as a promising modality for the treatment of prostate cancer.

With regards to the application of SDT in the treatment of cancer, a general protocol is applied, which involves the systemic administration of the sensitizer, either via blood circulation (intravenous infusion) or via the oral route (for aminolaevulinic acid, which is a biosynthetic precursor of the sensitizer protoporphyrin IX), prior to ultrasound exposure of the tumor, 24–48 h later. It has been suggested that tumor accumulation of the sensitizer occurs within the first hour post‐infusion and that the sensitizer is cleared from blood circulation within 24 h [5], when accumulation in the healthy parenchyma is minimal. Hence, after 24 h, the sensitizer‐containing cancerous mass can be safely exposed to ultrasound.

The efficacy of SDT relies heavily on the intratumoural accumulation and subsequent cellular uptake of sonosensitizers. Extensive research has been conducted to study the kinetics of intratumoural accumulation of sensitizing agents and towards the development of advanced drug delivery approaches to improve the delivery of sonosensitizers to target tumors [6, 7, 8, 9, 10]. However, limited research studies have focused on the intracellular accumulation and retention kinetics/profile of sonosensitizers, which are of major importance and have clinical implications in the design of an effective treatment plan in SDT.

The human ATP‐binding cassette superfamily G member 2 (ABCG2) transporter is a subset of large ABC protein superfamily. Many of these proteins are efflux transporters and have been linked to multidrug resistance in cancer [11, 12]. An extensive range of therapeutic molecules has been identified as substrates of ABCG2 transporter, including several porphyrin‐based sensitizers, such as chlorin e6, protoporphyrin IX, and pheophorbide. Several studies have reported the ABCG2‐mediated efflux of porphyrins and demonstrated that inhibition of ABCG2 transporter function can improve PDT efficacy by improving cellular retention of the sensitizer [13, 14, 15, 16]. In addition, in vivo studies with ABCG2 knock‐out mice have demonstrated increased accumulation of porphyrin sensitizers compared to wild‐type mice [17]. Therefore, the ABCG2 can be considered one of the main factors that can affect SDT efficacy, which is expected to be enhanced via inhibition of ABCG2 activity.

A variety of ABCG2 inhibitors have been studied, aiming to design cancer therapeutic regimens with increased chemotherapy effectiveness and improved drug absorption. Examples of compounds explored, thus far, include Febuxostat, Fumitremorgin C and analogues (such as Ko132 or Ko143), which can block efflux by either binding to the transporter, competing with substrates, or causing conformational changes in the transporter protein to disable it. Unfortunately, most of these agents lack clinical approval due to low specificity or toxicity issues. Lapatinib is a clinically approved kinase inhibitor that blocks two key tyrosine kinase receptors: HER2 (Human epidermal growth factor receptor 2) and EGFR (Epidermal growth factor receptor). It is commonly used to treat advanced, HER2‐positive breast cancers. It has been demonstrated that lapatinib directly binds to and inhibits the ABCG2 transporter, similarly to other inhibitors, like Ko143, subsequently blocking the cellular efflux of ABCG2 substrates. This process leads to increased intracellular concentrations of these substrates, enhancing their therapeutic effects, as shown in Figure 1. By inhibiting ABCG2, lapatinib can increase cellular retention of protoporphyrin IX, enhancing the diagnostic fluorescence and therapeutic efficacy of PDT in cancers like glioma [15, 18, 19]. The function of ABCG2 inhibition makes lapatinib a valuable candidate for its inclusion in combination therapies with other anticancer agents or sensitizers to overcome any transporter‐mediated drug resistance.

**FIGURE 1:**
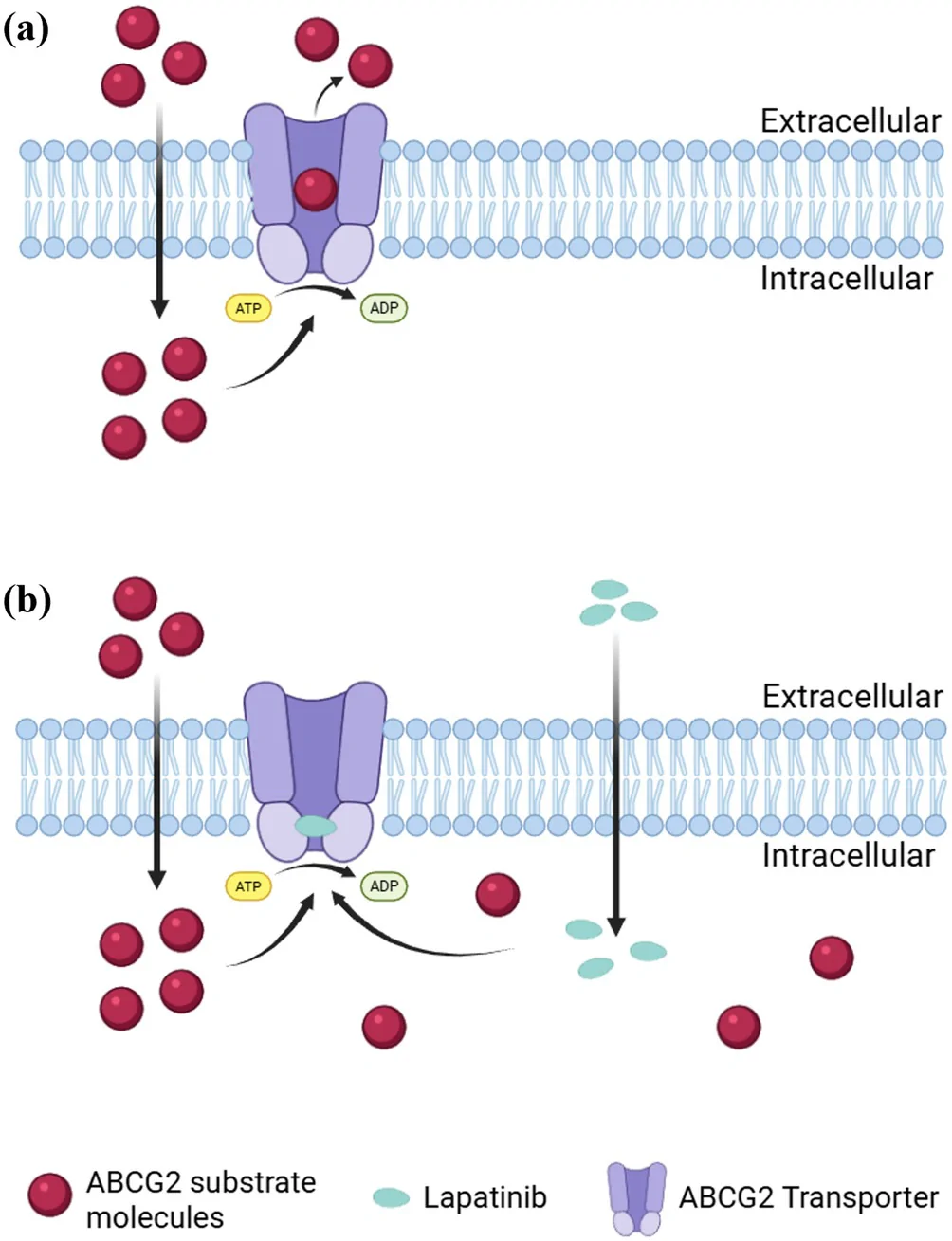
(a) ABCG2‐mediated drug efflux. (b) Lapatinib inhibition on ABCG2 efflux for intracellular drug concentration enhancement.

Prostate cancer manifests significant intratumoral and/or inter‐patient heterogeneity with respect to histo‐morphological presentation, as well as metabolic and molecular profile. A very potent regulator of this heterogeneity is hypoxia, which can dominate different regions throughout the cancerous mass when these regions become distant from blood supply. The rise of hypoxia can occur even at the early stages of tumor development. In addition, both hypoxia and prolonged androgen deprivation therapy (ADT) can induce neuroendocrine differentiation [20], leading to the development of cell populations with further altered molecular profile and response‐to‐therapy capacity. This progressively augmented heterogeneity can be reflected in the way tumor cells uptake, retain, and transport chemical agents, such as drugs and sensitizers.

In this work, the effect of lapatinib on the efficacy of sonodynamic therapy against prostate cancer cells was investigated. Studies were carried out on cellular systems conditioned in normoxia and hypoxia, as well as on cells with induced neuroendocrine phenotype. For the sonodynamic treatment of cells, a previously developed and well‐characterized formulation comprised of hematoporphyrin‐containing polymer‐based nanoparticles (HPNPs) was employed [21, 22].

## 2 Materials and Methods

### 2.1 Materials

RPMI 1640, phosphate buffered saline (PBS), dialysis tubing (MWCO: 8 kDa), GAPDH Polyclonal Antibody (Invitrogen, PA1‐987), Pierce BCA Protein Assay, 8% Bolt Bis‐Tris Plus Mini Protein Gel (1.0 mm, WedgeWell format, NW00080BOX), 1× Bolt MES SDS Running Buffer (LC5929), iBlot 2 Dry Blotting System, SuperSignal West Pico PLUS Chemiluminescent Substrate (34577), and ProLong Gold Antifade Mountant with NucBlue (P36981) were from Thermo Fisher Scientific, UK. ABCG2 Antibody (BXP‐21) was from Santa Cruz Biotechnology (sc‐58 222). Anti‐mouse IgG, HRP‐linked Antibody (7076P2) and Anti‐rabbit IgG, HRP‐linked Antibody (7074P2) were from Cell Signaling Technology. Anti‐Mouse IgG H&L (Alexa Fluor 488) preadsorbed was from Abcam (ab150117). Hematoporphyrin dihydrochloride, poly (L‐glutamic acid‐L‐tyrosine) 4:1, sodium salt (PGATyr) (MW: 20–50 kDa), polyvinyl alcohol (PVA, MW: 124 kDa), Ko143, Lapatinib, Dovitinib, 3‐(4,5‐dimethylthiazol‐2‐yl)‐2,5‐diphenyltetrazolium bromide (MTT), and dimethyl sulfoxide (DMSO) were from Sigma‐Aldrich, UK. Pheophorbide a was from Cambridge Bioscience, UK.

### 2.2 Cell Culture

The PC3 human‐derived prostate cancer cell line was obtained from the American Type Culture Collection (ATCC). The PC3 cell line was maintained in RPMI 1640 medium supplemented with 10% FBS and 1% penicillin & streptomycin (37°C, 5% CO_2_). Hypoxic PC3 cells were generated by incubating and passaging PC3 cells in hypoxia (37°C, O_2_ ≤ 1%, 5% CO_2_), using the same growth medium mixture, for 3 weeks. Neuroendocrine differentiated PC3 cells, here referred to as NEPC cells, were generated using a previously validated dovitinib‐induced neuroendocrine differentiation protocol. Briefly, PC3 cells were cultured (37°C, 5% CO_2_) in medium containing 6 μM of dovitinib for 3 weeks, and by replacing the differentiation medium with fresh once every 3 days. This protocol has been shown to induce neuroendocrine differentiation in PC3 cells, resulting in neurite‐like morphological changes, increased expression of neuroendocrine markers, and increased neuron‐specific enolase expression in PC3 xenograft tumors following systemic dovitinib treatment [23]. In this study, PC3 differentiation was also confirmed with western blot analysis (supporting information), using neuron‐specific enolase (NSE) as a differentiation marker (Figure S1).

### 2.3 Preparation of HPNPs

The preparation of HPNPs was adapted from a previously developed protocol [22]. In brief, 5 mg of HP (hematoporphyrin) was dissolved in 10 mL of DMSO. Ten milligrams of PGATyr was dissolved in deionized water and was added into the above solution. The resulting mixture was added dropwise into the 5 mL 0.5 mg/mL PVA solution. After constant stirring for 1 h at room temperature, the mixture was transferred into a dialysis tube (MWCO: 8 kDa) and dialyzed for 24 h. The suspension was then centrifuged at 40000 *g* for 1.5 h at 12°C using an Optima XPN‐100 Ultracentrifuge. The supernatant was discarded, and the precipitated pellet was resuspended in 2 mL deionized water. The suspension was sonicated for 3 min and then dialyzed overnight. After the dialysis, the suspension was sterile filtered through a 0.22 μm syringe filter. The suspension was then snap‐frozen with liquid nitrogen and freeze dried for 24 h. The dried sample was resuspended in PBS and was stored in the fridge, protected from light.

### 2.4 Intracellular Accumulation Assay for HPNP

Cells were seeded in wells of 96‐well plates at optimized density (2 × 10^4^ cells per well for normoxic PC3, 2.5 × 10^4^ cells per well for hypoxic PC3 cells, 3 × 10^4^ cells per well for NEPC cells) and incubated for 48 h. After 24 h, the culture medium was replaced with fresh medium. For each cell type, two identical 96‐well plates were prepared in parallel: one for fluorescence measurements and the other for assessment of metabolic activity using the MTT assay. Cells were incubated with HPNP‐containing medium in the presence or absence of lapatinib. Lapatinib was dissolved in DMSO, and control cells received an equivalent volume of DMSO vehicle. Cells were incubated for 2, 4, 8, or 24 h, after which fluorescence measurements and MTT assays were performed. For fluorescence analysis, the culture medium was removed, and cell monolayers were washed with PBS. DMSO was then added to each well to extract intracellular HPNP. Following incubation for 45 min in the incubator, the extracts were transferred to black, flat‐bottom 96‐well plates, and fluorescence was measured using an excitation wavelength of 534 nm and an emission range of 570–750 nm. Cellular metabolic activity was assessed in parallel using the MTT assay, and fluorescence values were normalized accordingly.

### 2.5 Intracellular Retention Assay for HPNP

Normoxic PC3, hypoxic PC3, and NEPC cells were seeded into 96‐well plates at optimized densities of 2 × 10^4^, 2.5 × 10^4^, and 3 × 10^4^ cells/well, respectively, and allowed to adhere for 24 h. For each cell type, two identical plates were prepared in parallel: one for fluorescence measurements and the other for assessment of metabolic activity using the MTT assay.

Cells were then incubated with HPNP‐containing culture medium in the presence or absence of lapatinib. The optimized HPNP concentrations (expressed as HP equivalents) were 20 μg/mL for normoxic PC3 cells, 15 μg/mL for hypoxic PC3 cells, and 25 μg/mL for NEPC cells. Lapatinib was dissolved in DMSO, and control cells received an equivalent volume of DMSO.

After 24 h of HPNP exposure, the treatment medium was removed and replaced with fresh growth medium containing either lapatinib or the corresponding volume of DMSO, but no HPNP. Cells were then incubated for a further 0, 2, 4, 8, or 24 h to assess intracellular HPNP retention. The 0 h samples were processed immediately following removal of the HPNP‐containing medium.

Fluorescence measurements and MTT assays were performed at each time point according to the procedures described in Section 2.4. Fluorescence values were normalized to cellular metabolic activity determined by the parallel MTT assay. Exported HP was not directly quantified in the culture medium, and the efflux level was inferred from the reduction in intracellular HP fluorescence rather than confirmed by direct secreted HP measurement.

### 2.6 Effect of Lapatinib on SDT Efficacy

Normoxic PC3, hypoxic PC3, and NEPC cells were seeded into 96‐well plates at optimized densities of 2 × 10^4^, 2.5 × 10^4^, and 3 × 10^4^ cells/well, respectively, and incubated for 24 h. Cells were subsequently treated with HPNP in the presence or absence of lapatinib. The optimized HPNP concentrations (expressed as HP equivalents) were 20 μg/mL for normoxic PC3 cells, 15 μg/mL for hypoxic PC3 cells, and 25 μg/mL for NEPC cells. Lapatinib was used at a final concentration of 2 μM for all cell lines. After 24 h of incubation, the treatment medium was removed, cells were washed with phosphate‐buffered saline (PBS), and fresh growth medium containing either lapatinib or vehicle control was added. Cells were then subjected to SDT using an SP100 Sonoporator (Sonidel Ltd., Ireland) operating at a frequency of 1 MHz, an acoustic intensity of 2.5 W/cm^2^, a 50% duty cycle, a pulse repetition frequency of 100 Hz, and an exposure time of 30 s. Ultrasound coupling gel was applied between the transducer and the underside of each well to ensure efficient acoustic coupling. Following SDT, cells were incubated for a further 24 h under their respective treatment conditions, after which cell viability was assessed using the MTT assay. The effects of SDT during both the HPNP accumulation and retention phases in the presence or absence of lapatinib were also evaluated. For the accumulation‐phase experiment, cells were incubated with HPNP with or without lapatinib for 3 h. The treatment medium was then removed, cells were washed with PBS, and fresh growth medium in the absence or the presence of lapatinib was added immediately before SDT treatment. For the retention‐phase experiment, cells were first incubated with HPNP with or without lapatinib for 24 h. Following removal of the treatment medium and washing with PBS, cells were incubated in fresh growth medium containing again in the presence or the absence of lapatinib for 2 h before SDT treatment. After sonication, cells were returned to the incubator for 24 h under their respective treatment conditions, and cell viability was determined using the MTT assay.

### 2.7 ABCG2 Western Blot

Whole‐cell lysates were prepared from normoxic PC3, hypoxic PC3, and NEPC cells, and protein concentrations were determined using the Pierce BCA Protein Assay. Lysates were diluted with 4× Bolt MES SDS Sample Buffer and RIPA lysis buffer to prepare 36 μL samples containing 20 μg of total protein. β‐Mercaptoethanol was added to a final concentration of 2.5% (v/v), and samples were denatured at 95°C for 10 min.

Proteins were separated by SDS‐PAGE using an 8% Bolt Bis‐Tris Plus Mini Protein Gel (1.0 mm, WedgeWell format) in 1× Bolt MES SDS Running Buffer. A 36 μL aliquot of each sample was loaded alongside 5 μL of SeeBlue Plus2 Prestained Protein Standard. Electrophoresis was performed at a constant current of 50 mA for 2 h. Proteins were then transferred onto a blotting membrane using the iBlot 2 Dry Blotting System.

Following transfer, proteins were fixed to the membrane by incubation in 4% paraformaldehyde (PFA) for 20 min. Membranes were washed with PBST and blocked in 5% bovine serum albumin (BSA) in PBST for 1 h at room temperature with gentle agitation. Membranes were incubated overnight at 4°C with anti‐ABCG2 primary antibody diluted 1:1000 in 3% BSA in PBST. After washing with PBST, membranes were incubated for 2 h at room temperature with HRP‐conjugated horse anti‐mouse IgG secondary antibody diluted 1:5000 in 3% BSA in PBST.

Membranes were subsequently washed, re‐blocked in 5% BSA in PBST for 1 h, and incubated overnight at 4°C with anti‐GAPDH primary antibody diluted 1:20000 in 3% BSA in PBST. Following further PBST washes, membranes were incubated for 2 h at room temperature with HRP‐conjugated goat anti‐rabbit IgG secondary antibody diluted 1:15000 in 3% BSA in PBST.

After a final series of PBST washes, protein bands were detected using SuperSignal West Pico PLUS Chemiluminescent Substrate and imaged using a ChemiDoc Imaging System (Bio‐Rad Laboratories, Hercules, CA, USA). Band intensities were quantified using ImageJ software.

### 2.8 Immunofluorescence for ABCG2

Normoxic PC3, hypoxic PC3, and NEPC cells were seeded onto sterile 13 mm glass coverslips in 24‐well plates at densities of 1 × 10^5^ cells/well for normoxic and hypoxic PC3 cells and 2 × 10^5^ cells/well for NEPC cells. Following 48 h of incubation, cells were washed with PBS and fixed with 4% PFA for 30 min at room temperature. Cells were then washed with PBS, permeabilized with 0.2% (v/v) Triton X‐100 for 30 min, and blocked with 5% BSA in PBST for 1 h at room temperature.

Cells were incubated overnight at 4°C with anti‐ABCG2 primary antibody diluted 1:100 in 1% BSA in PBST. Following PBS washes, cells were incubated for 2 h at room temperature, protected from light, with Alexa Fluor 488‐conjugated goat anti‐mouse IgG secondary antibody diluted 1:200 in 1% BSA in PBST. Coverslips were then washed with PBST and mounted onto glass microscope slides using ProLong Gold Antifade Mountant with NucBlue (Hoechst 33342). Mounted slides were protected from light and allowed to cure at room temperature for 24 h before imaging.

Fluorescence images were acquired using an Olympus fluorescence microscope.

### 2.9 ABCG2 Activity Level

Normoxic PC3, hypoxic PC3, and NEPC cells were seeded in 12‐well plates at densities of 3 × 10^5^, 3.5 × 10^5^, and 4 × 10^5^ cells/well, respectively. For each cell type, two identical plates were prepared in parallel: one for the ABCG2 substrate pheophorbide a (Pha) fluorescence measurements and the other for assessment of metabolic activity using the MTT assay.

Cells were incubated with Pha (1 μM) either alone or in combination with lapatinib for 3 h. Following incubation, cells treated with Pha alone were incubated in fresh culture medium, whereas cells in the combination group were incubated in fresh medium containing lapatinib (2 μM) for an additional 1 h. Cells were subsequently lysed, and the lysates were centrifuged to remove cellular debris. The resulting supernatants were collected, and Pha fluorescence was measured using excitation and emission wavelengths of 400 ± 2.5 nm and 677 nm, respectively.

Pha fluorescence values were normalized to cellular metabolic activity determined by the parallel MTT assay. ABCG2 transporter activity was expressed as the percentage change in normalized Pha fluorescence in cells treated with Pha plus lapatinib relative to cells treated with Pha alone.

### 2.10 Statistical Analysis

GraphPad Prism 9 was employed for the data presentation and analysis, unless otherwise stated. Statistical analysis of significance was performed using analysis of the means and comparison of data groups. The latter was performed using one‐way ANOVA with Tukey post‐test for column statistics and two‐way ANOVA with Bonferroni post‐test for grouped statistics. Error bars represent ± the standard error mean (SEM). A single asterisk (*) indicates *p* < 0.05, a double asterisk (**) indicates *p* < 0.01, and a triple asterisk (***) indicates *p* < 0.001.

## 3 Results

### 3.1 Effect of Lapatinib on the Intracellular Accumulation of HPNP

The effect of lapatinib on the cellular uptake and accumulation of HPNP nanoparticles in normoxic PC3, hypoxic PC3, and NEPC cells was evaluated. The internalization and accumulation of HPNP was determined by measuring HP fluorescence at different time points within 24 h, following the addition of HPNP, in the presence or the absence of lapatinib. As shown in Figure 2, a rapid increase in nanoparticulate HP accumulation was observed within the first 2 h of incubation with HPNP, either in the presence or absence of lapatinib. Thereafter, cellular uptake continued to rise over the 24 h incubation period, although at a slower rate compared to the initial 2 h. The data also demonstrated that within the 24 h incubation time, there was no significant effect of lapatinib on cellular uptake and accumulation of nanoparticulate hematoporphyrin (*p* > 0.05).

**FIGURE 2:**
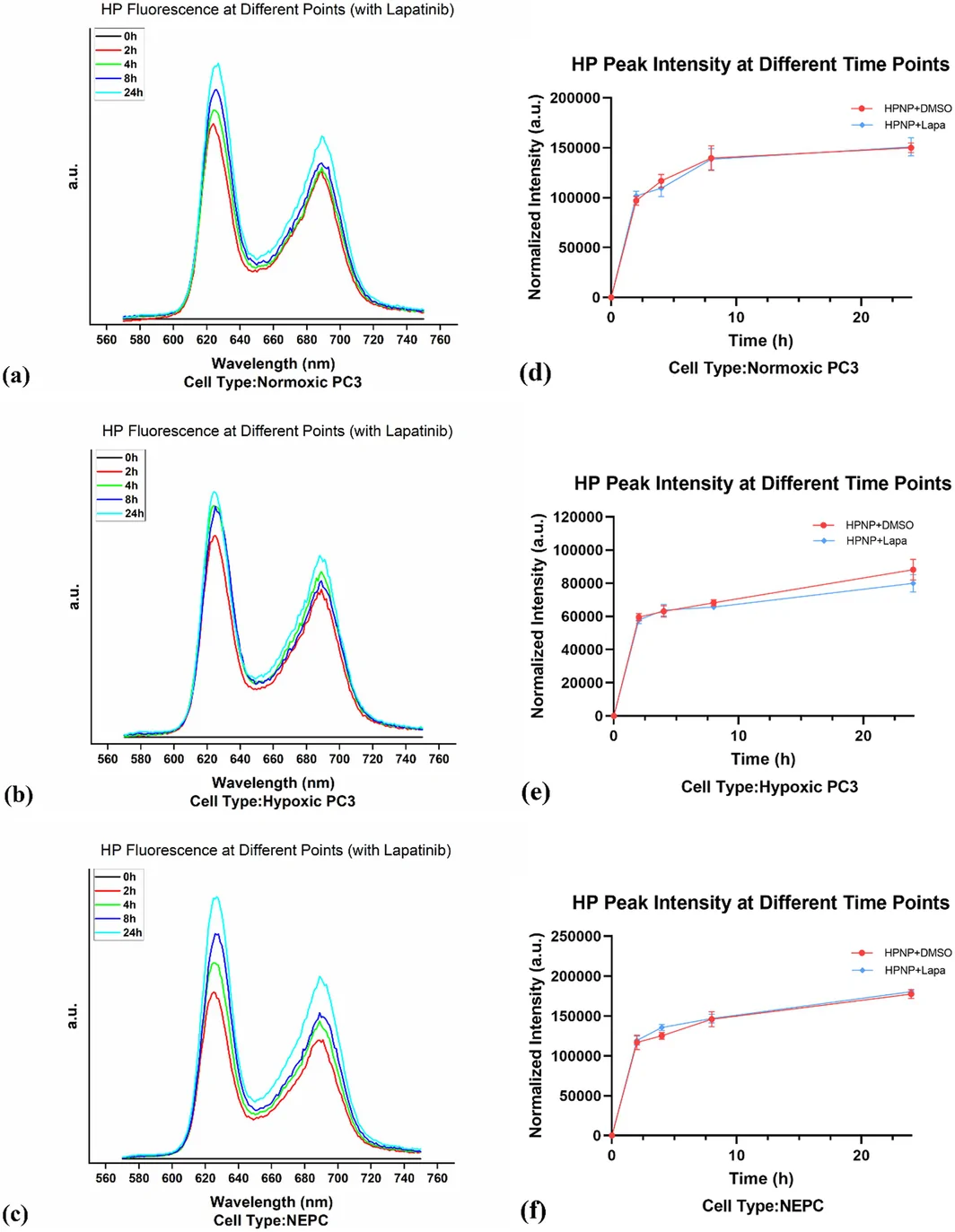
HP fluorescence spectra (average values) of (a) Normoxic PC3, (b) hypoxic PC3, and (c) NEPC cells after incubation with HPNPs‐containing cell culture medium plus lapatinib for 2 h, 4 h, 8 h, and 24 h. Curve graphs demonstrating intracellular HP fluorescence intensity changes with time at 625 nm of (d) normoxic PC3, (e) hypoxic PC, and (f) NEPC cells after incubation with HPNPs‐containing cell culture medium for 2, 4, 8, and 24 h with/without lapatinib added. ns, not significant; *p < 0.05, **p < 0.01, **** *p* < 0.0001 (*n* = 7).

### 3.2 Effect of Lapatinib on the Intracellular Retention of HPNP

The effect of lapatinib on the retention of nanoparticulate HP in PC3, hypoxic PC3 and NEPC, in the absence of HPNP in the incubation systems, was assessed. The retention of nanoparticulate HP was determined by measuring HP fluorescence at different time points within 24 h, following the removal of the HPNP from the incubation media, in the presence or the absence of lapatinib. As shown in Figure 3, the intracellular concentration of HP was almost halved within the first 2 h in all three cellular systems. More specifically, cellular HP was reduced by 60.20%, 46.87% and 69.25% for PC3, hypoxic PC3 and NEPC, respectively. Following the 2 h time point, the cellular retention of HP plateaued, with 12.62%, 24.52% and 12.91% of the sensitizer remaining in the cells at 24 h, with no detected retention improvement due to the presence of lapatinib (*p* > 0.05). The data demonstrate that lapatinib had no significant effect on nanoparticulate HP retention in normoxic PC3 cells at any time point throughout the 24 h incubation following removal of HPNP from the systems. For hypoxic PC3 cells, lapatinib was shown to improve retention by 9.80% (*p* = 0.004) at 2 h, 7.61% (*p* = 0.038) at 4 h and 8.23% at 8 h, after which point the effect on retention turned insignificant. Similarly, for NEPC cells, the agent improved HP retention by 5.09% and 5.52% at the 2 h (*p* = 0.03) and by 8 h time points (*p* = 0.028), respectively.

**FIGURE 3:**
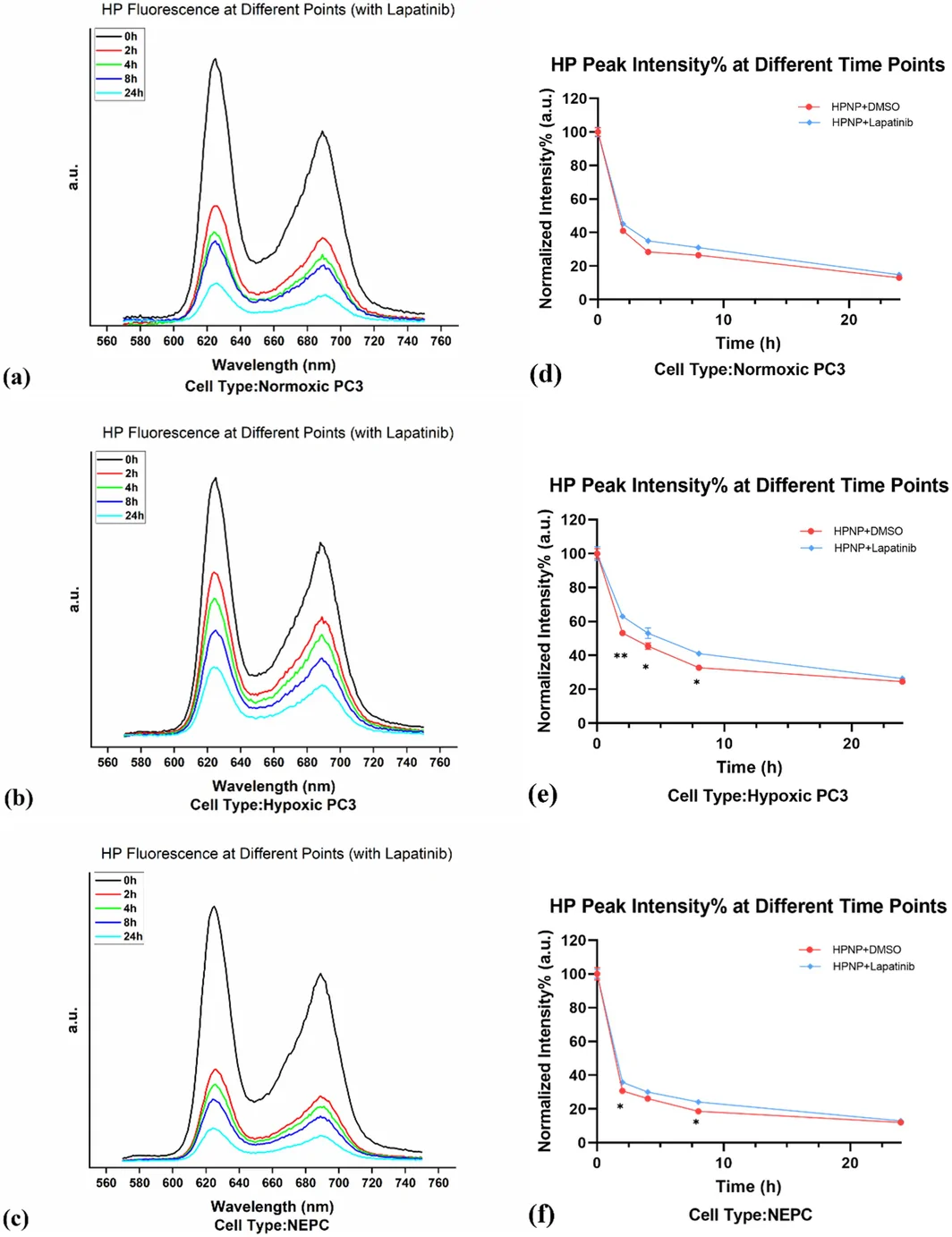
Averaged HP fluorescence spectra of (a) Normoxic PC3, (b) hypoxic PC3, and (c) NEPC cells after replacing HPNPs‐containing cell culture medium for 0 h, 2, 4, 8, and 24 h with lapatinib added. Curve graphs that demonstrate %intracellular HP fluorescence intensity changes with time at 625 nm of (d) normoxic PC3, (e) hypoxic PC, and (f) NEPC cells after replacing the HPNPs‐containing cell culture medium for 0 h, 2, 4, 8, and 24 h with/without lapatinib added. The % fluorescence intensity at 0 h after replacing the medium is set as 100%. ns, not significant; *p < 0.05, **p < 0.01 (*n* = 7).

### 3.3 The Effect of Lapatinib on SDT Efficacy

Based on the HPNP accumulation and retention profiles in the absence or the presence of lapatinib, the effect of the latter on SDT efficacy was investigated at different times during incubation and following incubation of the target cells with the nanoformulation. Data in Figure 4a demonstrate that, at the 3 h time point following incubation of the cellular systems with HPNP, lapatinib did not produce a statistically significant enhancement in SDT efficacy in normoxic PC3, hypoxic PC3, or NEPC cells. This result correlates with the nanoparticulate HP accumulation data (Figure 2) showing no significant lapatinib effect for three cell types. However, comparison of the response profiles suggests a tendency towards greater SDT‐induced cytotoxicity in hypoxic PC3 cells in the presence of lapatinib, whereas any corresponding effect in NEPC cells appeared limited. The efficacy of SDT was also examined 2 h after removing the HPNP from the cell systems. As shown in Figure 4b, lapatinib did not produce a statistically significant enhancement of SDT efficacy in either normoxic PC3 or hypoxic PC3 cells. However, a modest trend towards increased SDT efficacy was observed in hypoxic PC3 cells following lapatinib treatment. In NEPC cells, lapatinib improved SDT efficacy by a statistically significant 11.5%, while the synergy of the combination treatment did not exceed 0.28%.

**FIGURE 4:**
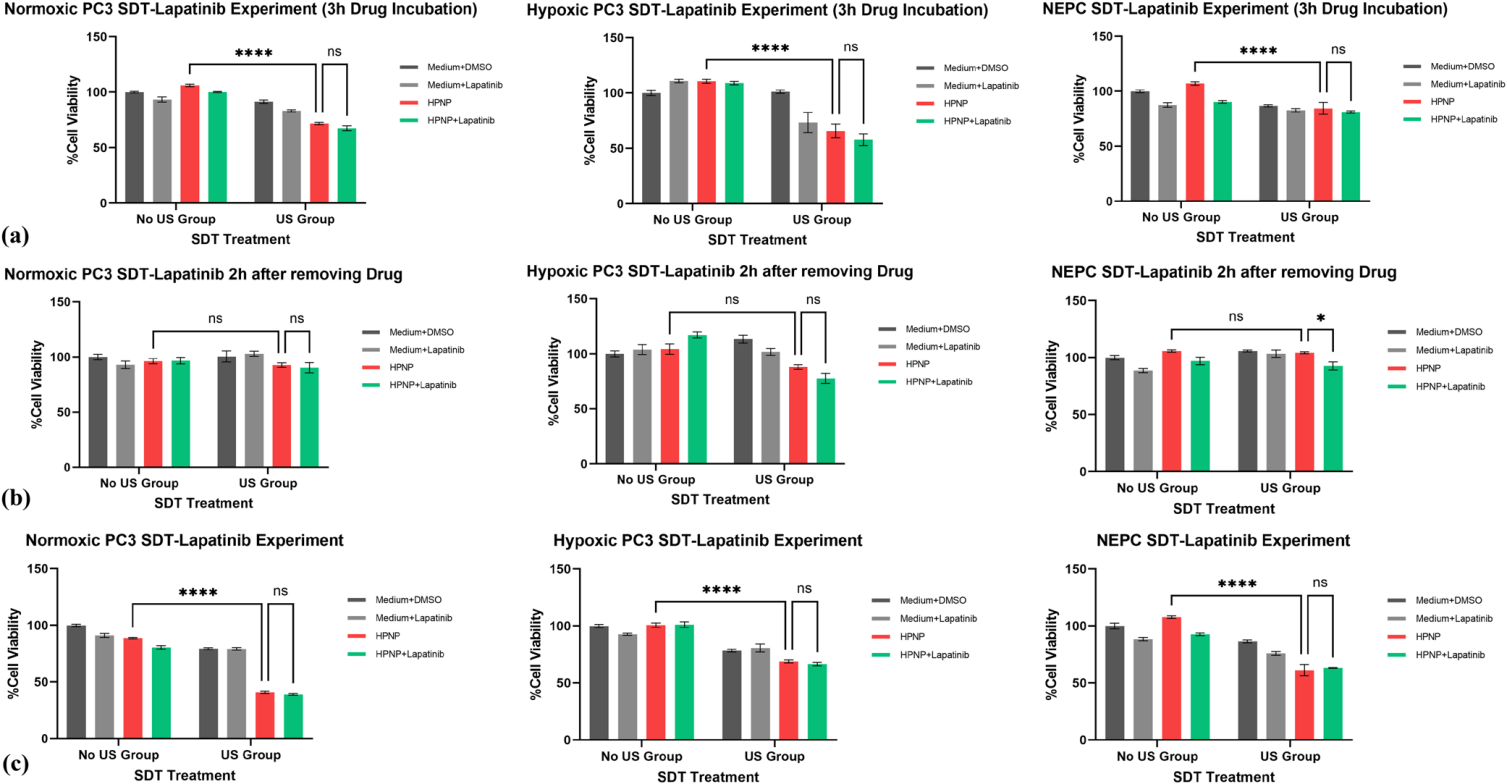
The % cell viability of SDT + lapatinib experiments on the three cell types: (a) SDT was performed when cells were incubated with HPNP‐containing cell culture medium + lapatinib for 3 h. (b) cells were incubated with HPNP‐containing cell culture medium + lapatinib for 24 h and SDT performed 2 h after replacing with agent‐free cell culture medium. (c) cells were incubated with HPNPs‐containing cell culture medium + lapatinib for 24 h and SDT was performed immediately after replacing with agent‐free cell culture medium. The concentration of lapatinib used was 2 μM. Values are displayed as mean ± SEM, ns, not significant; *p < 0.05, ****p < 0.0001 (*n* = 5).

Given that the protocol in most sonodynamic treatment studies on cellular systems includes the application of ultrasound 24 h after incubation of the systems with the sensitizing agent or the sensitizer‐carrying nanoparticles, the efficacy of SDT in the absence or the presence of lapatinib was also examined at the 24 h time point following incubation of the cellular systems with HPNP. Data in Figure 4c indicate that although SDT performance was overall improved compared to the 3 h incubation and 2 h retention timepoints, there was no effect of lapatinib on SDT efficacy for all target cell systems. The synergistic effects are summarized in Table 1.

**TABLE 1:** SDT synergy and lapatinib‐SDT synergy for normoxic PC3, hypoxic PC and NEPC cells.

	SDT synergy (US‐HPNP) = %SDT toxicity−%US toxicity−%HPNP toxicity			Lapatinib‐SDT synergy = %combo toxicity−%SDT toxicity−%lapatinib toxicity		
	3 h drug incubation	2 h after removing drug	24 h drug incubation	3 h drug incubation	2 h after removing drug	24 h drug incubation
Normoxic PC3	25.99%	4.00%	27.14%	−2.48%	−4.71%	−7.04%
Hypoxic PC3	46.18%	29.62%	10.29%	18.76%	14.17%	−5.04%
NEPC	9.46%	7.35%	31.68%	−8.98%	0.28%	−11.60%

### 3.4 ABCG2 Levels and Distribution

Immunofluorescence and Western blot analyses were performed for cell systems in order to investigate the effect of cellular nature and hypoxia on the expression and subcellular localization/distribution of ABCG2 transporters. Figure 5a,c indicate that ABCG2 transporters are distributed throughout the cytoplasmic space in normoxic PC3 and NEPC cells, while ABCG2 is accumulated in the cytoplasmic membrane in hypoxic PC3 cells, as depicted in Figure 5b.

**FIGURE 5:**
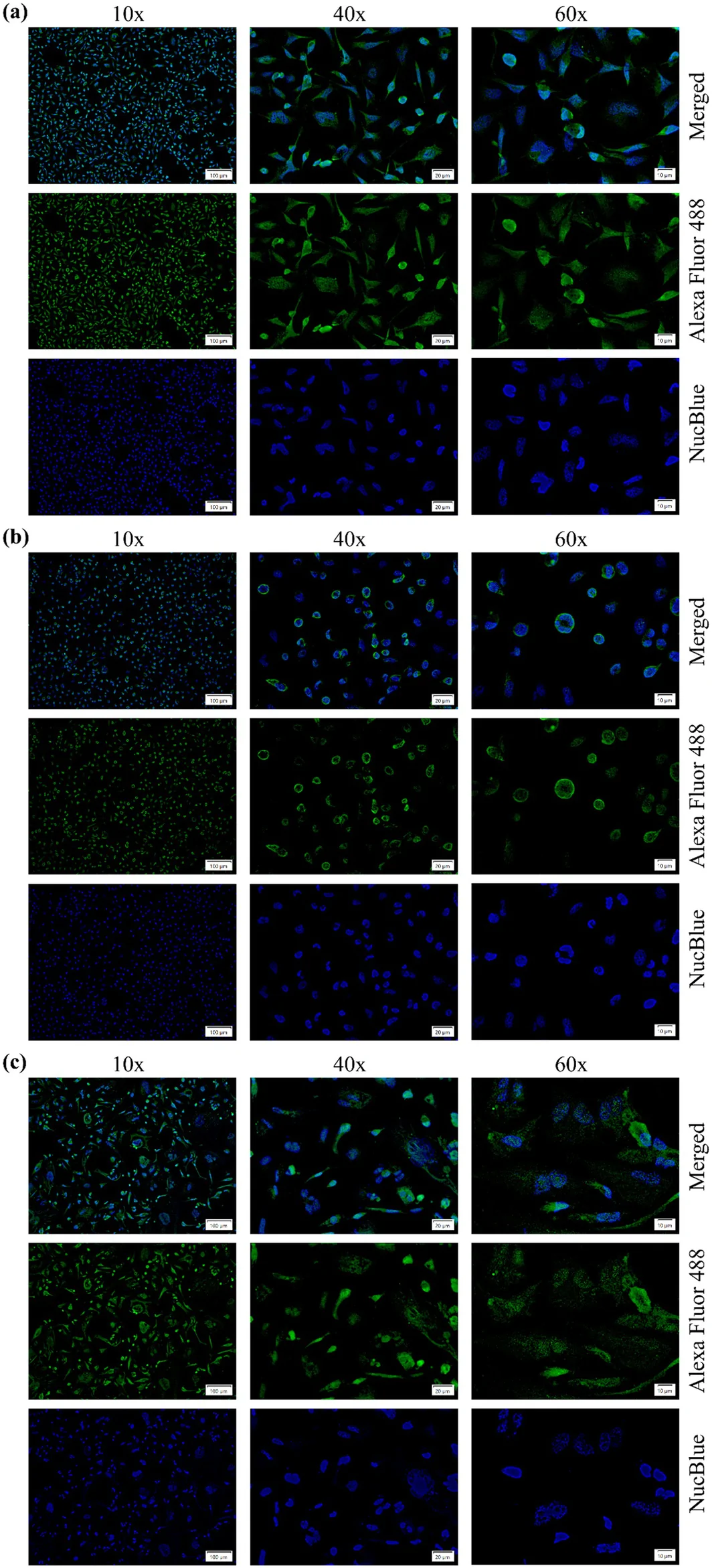
ABCG2 immunofluorescence images of (a) normoxic PC3, (b) hypoxic PC3 and (c) NEPC cell types. Cells were stained with the ABCG2 primary antibody, Alexa Fluor 488 secondary antibody and nuclei were stained with NucBlue. Distribution of ABCG2 is shown as green staining, with hypoxic PC3 cells showed preferential localization in the cytoplasmic membrane, while normoxic PC3 and NEPC show cytoplasmic distribution.

In addition to the immunofluorescence staining, Western blot analysis was also performed to determine cellular levels of ABCG2 protein. Figure 6a shows the distinct bands of ABCG2 in the three cell types, with observed molecular weights of ~65 kDa and ~72 kDa. The intensity of the bands was quantified and normalized to the housekeeping protein GAPDH, which showed consistent levels across all lines. Based on the quantification data presented in Figure 6b, the highest protein levels of ABCG2 on average were detected in normoxic PC3, followed by the protein levels in NEPC, while hypoxic PC3 cells were associated with the lowest average in ABCG2 protein levels (*p* > 0.05).

**FIGURE 6:**
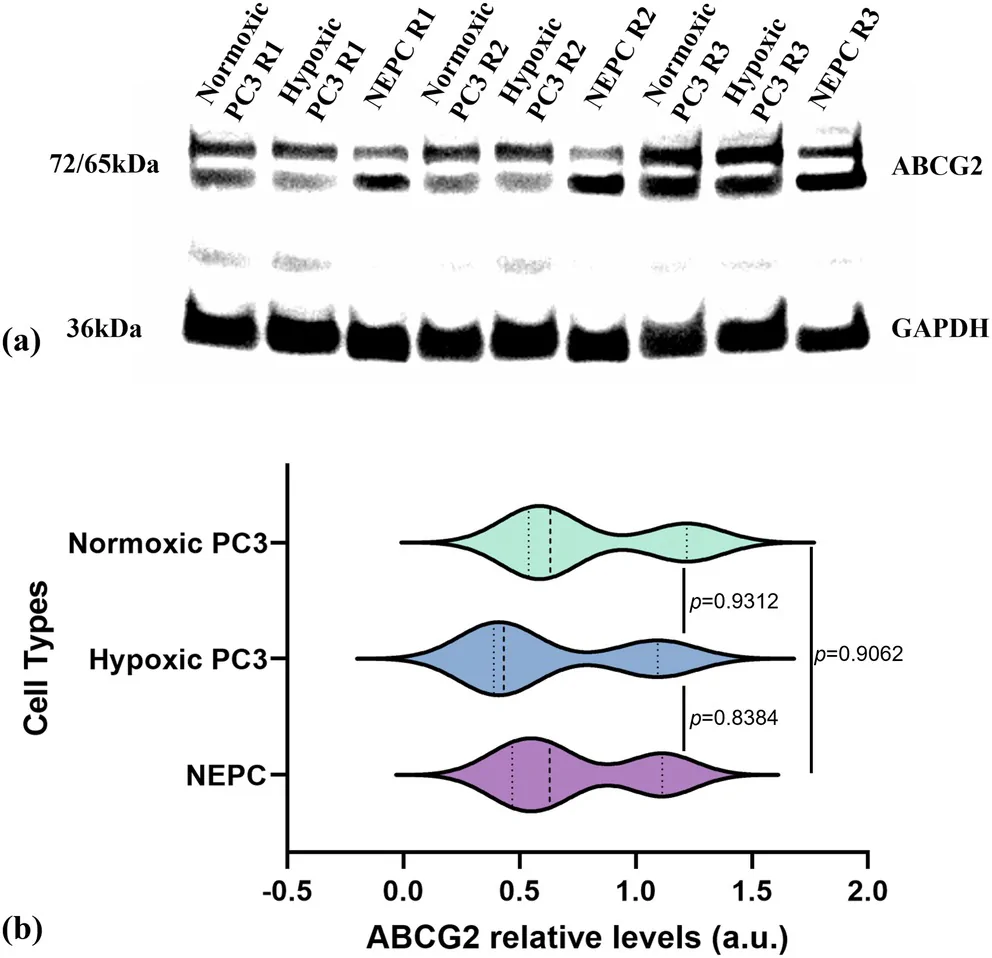
(a) Western blot results of ABCG2 relative cellular protein levels in normoxic PC3, hypoxic PC3 and NEPC cells, with R indicating the replicate sample. ABCG2 bands at ~65 kDa and ~72 kDa. GAPDH was used as the housekeeping protein at ~36 kDa. (b) Violin plots show the probability density distribution of ABCG2 protein level (normalized to GAPDH) across normoxic PC3, hypoxic PC3 and NEPC cells. The width of each violin shape represents the kernel density estimate of the data. Inside each violin, dashed line represents the median, while dotted lines represent the 25th and 75th percentiles (*n* = 3).

### 3.5 ABCG2 Activity

Apart from the ABCG2 transporter expression levels and subcellular distribution, the functionality/activity of ABCG2 transporters can also influence the porphyrin accumulation and retention profiles among different cell systems. Therefore, the activity of ABCG2 in the target cell systems was determined and the results are shown in Figure 7. Although no statistical difference was observed in ABCG2 activity among the cellular systems, hypoxic PC3 cells exhibited the highest ABCG2 activity, whereas normoxic PC3 cells showed the lowest ABCG2 activity. The trend of response to SDT in the presence of lapatinib appeared to show a positive association with ABCG2 activity in the current cellular systems.

**FIGURE 7:**
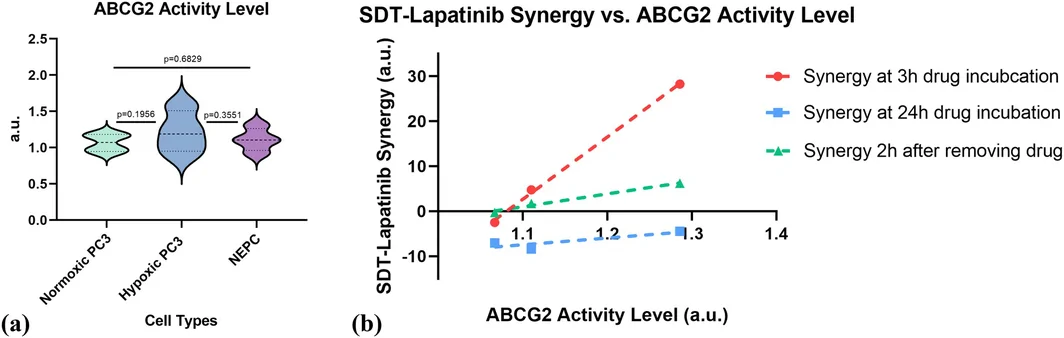
(a) Violin plots show the probability density distribution of ABCG2 activity across normoxic PC3, hypoxic PC3, and NEPC cells. The width of each violin shape represents the kernel density estimate of the data. Inside each violin, dashed line represents the median, while dotted lines represent the 25th and 75th percentiles (*n* = 4). (b) SDT‐Lapatinib synergy correlation with ABCG2 activity based on results from normoxic PC3, hypoxic PC3, and NEPC cells.

## 4 Discussion

This study explored the effect of lapatinib on sonodynamic therapy (SDT) efficacy based on the previously reported capacity of this agent to regulate the cellular efflux of porphyrins via inhibition of the ABCG2 transporters. The study employed hematoporphyrin‐carrying nanoparticles (HPNP), as distinct from free hematoporphyrin, given that current trends are focused on the nanoparticle‐assisted delivery of sensitizing agents in SDT. The HPNP formulation has been previously well‐characterized with regards to its cellular uptake, the in vivo biodistribution profile and kinetics, and its SDT efficacy for the treatment of PC3 cells [21, 22].

The cellular accumulation and retention of a therapeutic agent are critical for the efficacy of multimodal treatments such as SDT, as they influence the optimal timing of ultrasound exposure. Cellular uptake and efflux of agents are dynamic processes regulated by cell type, substance properties, the extracellular and intracellular environment, and the substance concentration [24]. In this study, nanoparticulate HP was rapidly taken up by all three cellular systems within the first 2 h after adding the nanoparticles, followed by a slower uptake for up to 24 h. Lapatinib did not affect nanoparticulate HP accumulation during incubation. Although it was previously reported that in glioma cells treated with 5‐aminolevulinic acid (ALA), lapatinib significantly enhanced protoporphyrin IX accumulation [15], the system differs from the current one in both cell type and sensitizer generation/release pathway, which may alter the balance between uptake and efflux. Interestingly, the effect of lapatinib on the cellular accumulation of molecularly formed porphyrins has not been previously investigated. A recent breast cancer study combining chlorin e6 (Ce6) with lapatinib in a nanoplatform did not address ABCG2‐related synergy or sensitizer retention kinetics [25]. In the present study, lapatinib was used at the maximum concentration associated with minimal cytotoxicity, allowing its effect on sensitizer accumulation/retention and SDT efficacy to be examined with limited confounding by its kinase‐inhibitory toxicity. Although PC3 cells exhibit low to moderate levels of ABCG2, their use allowed evaluation of lapatinib effects across biologically distinct states, including hypoxic and neuroendocrine‐differentiated populations relevant to advanced and drug‐resistant prostate tumors [26, 27].

Sensitizer efflux and retention kinetics are also important when considering the timing of ultrasound exposure in clinical SDT. The two‐dimensional cellular (monolayer) system used here offers a simplified system to study the sensitizer accumulation and retention kinetics, bypassing the site‐dependent variables of diffusion and lymphatic drainage. In an In vivo/clinical setting, sensitizer accumulation and retention within the tumor microenvironment are less sharply defined. During the first 2–8 h following systemic administration, the sensitizer is expected to remain present in blood circulation, representing a period of active tumor accumulation [28]. Early ultrasound exposure may preferentially damage tumor vasculature, whereas longer intervals may favor tissue diffusion and cellular uptake. Accordingly, most clinical and preclinical In vivo studies apply ultrasound at 6–24 h after sensitizer administration to optimize tumor cell accumulation of the sensitizing agent, maximizing the therapeutic effect, while minimizing damage to healthy surrounding tissue and tumor vasculature [5, 21, 22]. Interestingly, studies in photodynamic therapy suggesting that sensitizer concentration in plasma, indicative of endothelial cell exposure, is more accurately correlated with an improved therapeutic effect than the intratumoral sensitizer concentration [29, 30]. This may reflect not only cellular uptake within the tumor, but also vascular damage and ischemic effects that contribute to tumor growth suppression. In vivo biodistribution studies of the HPNP formulation showed rapid tumor accumulation, reaching maximum levels 15 min after intravenous administration, with a sustained fluorescence signal over time, even after 24 and 48 h. However, tumor fluorescence does not distinguish extracellular from intracellular sensitizer, making the separate contribution of accumulation and retention difficult to define In vivo. Given the intratumoral heterogeneity and dysfunctional lymphatic system, the nanoparticulate HP accumulation and efflux are also likely to remain dynamic across the tumor mass.

The findings here showed no effect of lapatinib on the cellular accumulation of nanoparticulate HP, while the cell systems were being exposed to the HPNP suspension, that is, under constant supply of HPNP. It was also demonstrated that, following drug withdrawal, a sharp decrease in intracellular HP level was observed within the first 2 h in all three cell populations. This phase is likely to be dominated by passive diffusion and ABCG2‐mediated efflux [31, 32, 33, 34]. However, in the lapatinib‐treated groups, particularly in hypoxic PC3 cells, the decrease phase was significantly attenuated during the 2–8 h window. This highlights that lapatinib can extend the intracellular presence of the sonosensitizer, effectively prolonging the therapeutic window for performing SDT. This also underlines the value of lapatinib in improving the sensitizer intracellular levels in hypoxic areas of the tumor mass, where cell populations overexpress ABCG2 transporters and the sensitizer supply is expected to be low, due to the low vascularization levels [35].

The current findings suggest that lapatinib may contribute to enhanced SDT‐induced cytotoxicity under selected conditions, in association with improved intracellular retention of the sensitizing agent. Overall, treatment with lapatinib improved the mean SDT response compared with HPNP alone; however, this difference was not statistically significant. Although very limited statistically significant enhancement in SDT efficacy was observed at the tested time points, the response profiles indicated a tendency towards greater SDT effect in hypoxic PC3 cells at earlier treatment windows, particularly at 3 h of incubation with the HPNP formulation and 2 h following HPNP removal. These observations indicate that lapatinib may offer benefit primarily in cellular populations with higher ABCG2 activity, such as hypoxic populations, rather than facilitating a uniform enhancement across all prostate cancer cell states within a tumor. Although the efficacy of SDT after 3 h of exposure of the cellular systems to HPNP is similar to that after 24 h of exposure, lapatinib has no effect on SDT performance at the latter test time point group. It has been suggested that subcellular localization plays a considerable role in the efficacy of SDT [36]. The use of a nanoparticulate formulation for carrying the sensitizer would involve subcellular processing and certain intracellular localization kinetics, a process that may last several hours after exposing the cellular systems to the HPNP. Although the nanoparticulate HP cellular levels do not fluctuate after the first 2 h of HPNP exposure, the subcellular localization profile, and consequently, the sensitizer efflux‐related intracellular interactions, may differ. This may explain why only an early tendency towards improved SDT efficacy was observed in hypoxia.

Whether lapatinib would be beneficial in combination with SDT in a clinical setting is also likely to depend on tumor heterogeneity. Advanced prostate cancers are expected to contain both normoxic and hypoxic regions, with temporally and spatially variable exposure to nanoparticles and other agents. Tumors exposed to prolonged androgen deprivation therapy may also contain neuroendocrine‐like populations [37]. These factors are expected to influence how much ABCG2‐mediated efflux limits sensitizer retention and how lapatinib modulates the treatment response.

ABCG2 subcellular localization, protein level and transporter activity are known to be influenced by environmental conditions such as hypoxia. In the current study, although total cellular ABCG2 protein levels were not significantly different among the three cell types, ABCG2 subcellular localization and transporter activity profiles varied. Immunofluorescence studies showed that ABCG2 was distributed throughout the cytoplasm in normoxic PC3 and NEPC cells, whereas in hypoxic PC3 cells it showed preferential localization in the cytoplasmic membrane, a potentially important finding for efflux dynamics. This observed redistribution may reflect a functional adaptation of cancer cells to the hypoxic environment, potentially impacting drug efflux kinetics and subcellular drug partitioning. Importantly, hypoxia‐inducible factors (HIFs), particularly HIF‐1α, are known to modulate the trafficking and transcription of efflux transporters, including ABCG2 [38, 39]. Moreover, hypoxic PC3 cells had the highest ABCG2 transporter activity, while normoxic PC3 cells had the lowest ABCG2 transporter activity level, which was inversely correlated with the cellular protein levels determined with Western blotting. One possible explanation is that strong membrane association reduces extraction and transfer efficiency of transmembrane proteins during Western blotting [40]. Essentially, this aspect can explain the lower ABCG2 protein levels measured in hypoxic PC3 cells compared to the normoxic population. This underlines the importance of assessing transporter activity, as distinct from solely protein cellular levels, when predicting drug‐dependent therapeutic response. Here, the tendency towards improved response with lapatinib in hypoxia appears to be induced by the inhibition of active sensitizer efflux, with less dependence on the overall cellular ABCG2 protein level. This corroborates previous findings that lapatinib activity is strongly associated with ABCG2‐binding rather than the modification of gene or protein expression [15, 41, 42].

## 5 Conclusions

The current study demonstrates the potential of lapatinib to enhance the retention of nanoparticulate HP in prostate cancer cells through the inhibition of ABCG2 transporters. The enhancement is particularly pronounced under hypoxic conditions. These findings suggest that modulation of sensitizer retention may influence SDT response under selected conditions, highlighting the critical role of drug efflux regulation in this therapeutic modality. This work lays the foundations for repurposing the clinical use of lapatinib as a tyrosine kinase inhibitor in oncological treatment, including it in a rationalized combination with SDT. Further studies are required to refine the dose of lapatinib for achieving beneficial outcomes with regard to sensitizer efflux inhibition and subsequent augmentation of SDT effects.

## Author Contributions

**Yidong Wang:** investigation, methodology, validation, writing – original draft, formal analysis, data curation. **Hamzah Masood:** methodology, investigation. **Nikolitsa Nomikou:** conceptualization, investigation, methodology, visualization, writing – review and editing, project administration, supervision, resources.

## Funding

Open access funding provided by University College London within transformative agreements.

## Conflicts of Interest

The authors declare no conflicts of interest.

## Supporting information


**Figure S1:** NSE protein levels, quantified using Western blot, and normalized to GAPDH.

## Data Availability

The data that support the findings of this study are available on request from the corresponding author. The data are not publicly available due to privacy or ethical restrictions.
