# Supplementary material for: Investigating the Effect of Lapatinib on the Performance of Sonodynamic Therapy Against Prostate Cancer via Inhibition of the ABCG2 Efflux System
Source: Pharmacol Res Perspect. 2026 Oct 22;14(6):e70340. doi: 10.1002/prp2.70340 (PMC13691173; doi:10.1002/prp2.70340)
Supplement: Supplementary file 1 [file PRP2-14-e70340-s001.docx]

**Supplementary Information**

**Confirming dovitinib-induced PC3 cell neuroendocrine differentiation using Western Blot analysis for Neuron-Specific Enolase (NSE)**

**Method**

Whole lysates of PC3 and NEPC cells were produced, and their protein content was measured. The samples were then diluted to obtain an equal protein content (20μg) using 4X Bolt™ LDS sample buffer (Thermofisher, UK) containing 1.43M ß-mercaptoethanol (ß-ME) (Merck, UK) and excess cell lysis buffer. The samples were treated at 95°C for 10 min and then centrifuged at 14,000g for 10 min to collect the supernatant. The proteins were separated into an 8% SDS-PAGE gel and then transferred to a PVDF membrane (Thermofisher, UK). The separated protein samples were fixed onto the PVDF membranes using 4% paraformaldehyde for 30 min at room temperature and washed with PBST (PBS with 0.1% Tween® 20) to remove any excess PFA. Membranes were then blocked with 5% Bovine Serum Albumin (BSA) in PBST for 1.5h and then incubated with the primary antibody for GAPDH (Invitrogen, UK, PA1-987), diluted to 1:4000 with PBST 3% BSA, initially overnight at 4℃ and then for another 2h at room temperature with gentle shaking. Membranes were then washed with PBST and were blocked again with 5% BSA in PBST for 1.5h. The membranes were probed with the anti-NSE primary antibody (Invitrogen, UK, PA5-27452), diluted to 1:2500 in 3% BSA, initially overnight at 4℃ and then for another 2h at room temperature with gentle shaking. After washing with PBST, the membrane was probed with anti-rabbit IgG HRP-linked antibody (Cell Signalling Technology, UK, 7074P2) diluted to 1:2000 in PBST with 3% BSA, for 2h. The membrane was washed again and was left in PBST until visualisation. The visualisation solution (SuperSignal™ West Dura Extended Duration Substrate –Thermofisher, UK) was prepared by mixing equal parts of the substrate and stable peroxide components. The membrane was incubated with10 mL of the visualization solution at room temperature, for 5 min. The membrane was then imaged using the ChemiDoc imaging system (Bio-Rad Laboratories, Inc.). Images were analysed using ImageJ software, where the band intensity of the protein of interest (NSE) was normalised to the band intensity of GAPDH of each sample after removal of the background.

For comparative purposes and to further confirm the dovitinib-induced neuroendocrine nature of NEPC cells, the NSE protein level of LASCPC-01 (ATCC, USA), an established neuroendocrine prostate cancer cell line, was analysed with Western Blot, alongside with PC3 cells and NEPC cells.

**Results**

Data in Figure S1 demonstrate that NEPC cells display higher NSE protein levels than PC3 and LASCPC-01 cells. NSE is a strong neuroendocrine marker, and the data confirm that the dovitinib-induced differentiation of PC3 cells into cells with neuroendocrine phenotype was successful. Moreover, the fact that NSE protein levels in NEPC cells exceeded those of LASCPC-01 cells demonstrates that the neuroendocrine phenotype of NEPC cells is stronger than that of an established neuroendocrine prostate cancer cell line.

**Figure S1**: NSE protein levels, quantified using Western blot, and normalised to GAPDH
